# Impact of HIV-1 CRF55_01B infection on the evolution of CD4 count and plasma HIV RNA load in men who have sex with men prior to antiretroviral therapy

**DOI:** 10.1186/s12977-021-00567-z

**Published:** 2021-08-16

**Authors:** Lan Wei, Hao Li, Xing Lv, Chenli Zheng, Guilian Li, Zhengrong Yang, Lin Chen, Xiaoxu Han, Huachun Zou, Yanxiao Gao, Jinquan Cheng, Hui Wang, Jin Zhao

**Affiliations:** 1 Department of HIV/AIDS Control and Prevention, Shenzhen Center for Disease Control and Prevention, Shenzhen, China; 2 Key Laboratory of AIDS Immunology, Ministry of Health, Department of Laboratory Medicine, First Hospital of China Medical University, Shenyang, China; 3 School of Public Health (Shenzhen), Sun Yat-sen University, Shenzhen, China; 4 Kirby Institute, University of New South Wales, Sydney, Australia; 5 HKU-AIDS Institute Shenzhen Research Laboratory, Shenzhen Key Laboratory of Infection and Immunity, Guangdong Key Laboratory of Emerging Infectious Diseases, Third People’s Hospital of Shenzhen, Shenzhen, China

**Keywords:** Disease progression, Circulating recombinant form, CD4 count, Viral load

## Abstract

**Background:**

CRF55_01B is a newly identified HIV-1 circulating recombinant form originated from MSM in China. However, its impact on the disease progression and transmission risk has not been investigated. This study aimed to determine the impact of CRF55_01B infection on viral dynamics and immunological status so as to provide scientific evidence for further control and prevention effort on CRF55_01B. Linear mixed effect models were applied to evaluate CD4 cell count decline and viral load increase by subtype.

**Results:**

Of the 3418 blood samples, 1446 (42.3%) were CRF07_BC, 1169 (34.2%) CRF01_AE, 467 (13.7%) CRF55_01B, 249 (7.3%) type B, and 87 (2.5%) other subtypes (CRF_08BC, CRF_01B, C). CRF55_01B had become the third predominant strain since 2012 in Shenzhen, China. CRF55_01B-infected MSM showed lower median of CD4 count than CRF07_BC-infected MSM (349.5 [IQR, 250.2–474.8] vs. 370.0 [IQR, 278.0–501.0], P < 0.05). CRF55_01B infection was associated with slower loss of CD4 count than CRF01_AE (13.6 vs. 23.3 [cells/µl]¹/²/year, P < 0.05)among MSM with initial CD4 count of 200–350 cells/µl. On the other hand, those infected with CRF55_01B showed higher median plasma HIV RNA load (5.4 [IQR, 5.0–5.9]) than both CRF01_AE (5.3 [IQR, 4.8–5.7], P < 0.05) and CRF07_BC (5.0 log10 [IQR, 4.5–5.5], P < 0.001) at the initiation of antiretroviral therapy. Furthermore, the annual increasing rate of viral load for CRF55_01B infection was significantly higher than that of CRF07_BC (2.0 vs. 0.7 log10 copies/ml/year, P < 0.01).

**Conclusions:**

The relatively lower CD4 count and faster increase of plasma HIV RNA load of CRF55_01B-infected MSM without antiretroviral therapy suggest that CRF55_01B may lead to longer asymptomatic phase and higher risk of HIV transmission. Strengthened surveillance, tailored prevention strategies and interventions, and in-depth research focusing on CRF55_01B are urgently needed to forestall potential epidemic.

**Supplementary Information:**

The online version contains supplementary material available at 10.1186/s12977-021-00567-z.

## Introduction

HIV has very high genetic variability and diversity. To date, two main types, nine subtypes, 89 HIV circulating recombinant forms (CRFs) and multiple unique recombinant forms (URFs) had been recognized worldwide [1]. In Asia, the predominant genotypes are subtype B and C, CRF01_AE and their recombinants [2]. With the increasing trend of HIV prevalence among men who have sex with men (MSM) in China in the past decade [3], the three predominant genotypes co-circulated and rapidly mixed, leading to inevitable generation of new CRFs. Previous studies implied that CRFs accounted for more than 90% of all HIV-1 infection in China, and new CRFs played an increasing role in the nationwide or regional HIV pandemic [4, 5].

CRF55_01B, a CRF derived from CRF01_AE and B and circulating predominantly among MSM, was identified in 2012 in China [3]. Surveillance data indicated that CRF55_01B has been the third important epidemic CRFs since CRF07_BC and CRF08_BC were discovered in China in the 1990 s [3], and has brought about an outbreak among MSM in Shenzhen in 2013 [6]. Previous studies implied the origin time of CRF55_01B in China was around 2001, and first infected case was found in Shenzhen in 2007 [6]. Existing literature indicated that the prevalence of HIV-1 CRF01_AE and subtype B in Shenzhen had gradually decreased from 50% and 37.5% to 32.3% and 5.7% from 2006 to 2012, respectively. In contrast, the prevalence of CRF07_BC and CRF55_01B had rapidly increased from 25% to 0% in 2006 to 43.2% and 16.0% in 2012, respectively [7].

HIV-infected individuals without antiretroviral therapy are clinically characterized by loss of CD4 T-cell and rise of plasma HIV RNA load, which could result in increased risks for opportunistic infections and development of AIDS and AIDS-related deaths. Thus, both CD4 T-cell counts and plasma HIV RNA are important prognostic markers of progression to AIDS and serve as main indicators for initiation of antiretroviral therapy. Clinical studies revealed that HIV-1 subtype was closely related to HIV transmission and disease progression [8–11]. As a newly emerging strain, the evolutionary characteristics of CRF55_01B has been identified; nevertheless, its impact on the evolution of CD4 count and plasma HIV RNA load, has not been documented. In the present study, we aimed to determine the impact of CRF55_01B infection on viral dynamics and immunological status so as to provide reference for future control and prevention effort.

## Results

### Demographic characteristics

**Fig. 1:**
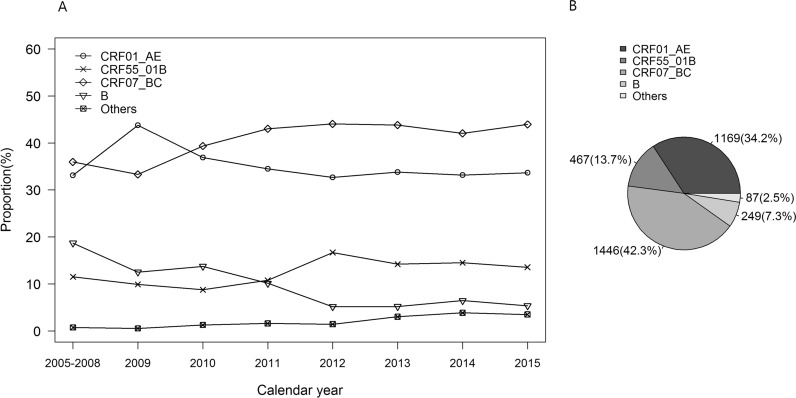
Genotype distribution of HIV-1 among the infected MSM in Shenzhen, China, 2005–2015

From 2005 to 2015, blood samples were collected from 3418 MSM at diagnosis of HIV infection in Shenzhen, with genotype distribution as follows: 1446 (42.3%) CRF07_BC, 1169 (34.2%) CRF01_AE, 467 (13.7%) CRF55_01B, 249 (7.3%) subtype B, and 87 (2.5%) other subtypes (CRF_08BC, CRF_01B, C, and others) (Fig. 1B). Of note, we found the distribution of CRF55_01B has a significant trend of proportions during the study years (χ^2^ = 4.12, P value < 0.05) and had become the third predominant strain since 2012 in Shenzhen, China (Fig. 1A). By excluding the other subtypes and the infections which were more than one year prior to sampling, there were 1792 recent infection for CRF07_BC, CRF01_AE, and CRF55_01B. Demographic characteristics of the 1792 MSM were shown in Table 1.

**Table 1:** Demographic characteristics of the 1792 MSM with recent infection of HIV-1

	CRF01_AE	CRF55_01B	CRF07_BC	P value
	N = 669	N = 282	N = 841	
Year				
2005–2008	26 (3.9)	9 (3.2)	22 (2.6)	0.179
2009	47 (7.0)	8 (2.8)	34 (4.0)	
2010	19 (2.8)	8 (2.8)	29 (3.4)	
2011	63 (9.4)	19 (6.7)	68 (8.1)	
2012	124 (18.5)	63 (22.3)	167 (19.9)	
2013	163 (24.4)	74 (26.2)	225 (26.8)	
2014	137 (20.5)	60 (21.3)	161 (19.1)	
2015	90 (13.5)	41 (14.5)	135 (16.1)	
Age (year)				
< 25	230 (34.4)	66 (23.4)	288 (34.2)	< 0.01
26–35	292 (43.6)	122 (43.3)	377 (44.8)	
36–45	105 (15.7)	58 (20.6)	136 (16.2)	
> 46	42 (6.3)	36 (12.8)	40 (4.8)	
Marital status				
Unmarried	542 (81.0)	178 (63.1)	654 (77.8)	< 0.01
Married	88 (13.2)	71 (25.2)	120 (14.3)	
Divorced or widowed	29 (4.3)	25 (8.9)	61 (7.3)	
Unknown	10 (1.5)	8 (2.8)	6 (0.7)	
Ethnicity				
Han	31 (4.6)	18 (6.4)	47 (5.6)	0.505
Non-han	638 (95.4)	264 (93.6)	794 (94.4)	
Education				
Below senior high school	209 (31.2)	114 (40.4)	254 (30.2)	< 0.05
Senior high school or technical secondary school	247 (36.9)	98 (34.8)	306 (36.4)	
College or university	210 (31.4)	67 (23.8)	275 (32.7)	
Unknown	3 (0.4)	3 (1.1)	6 (0.7)	
Census registration*				
Shenzhen resident	44 (9.9)	19 (10.3)	53 (9.7)	0.523
Temporary resident	289 (64.8)	126 (68.1)	381 (69.5)	
Floating or unknown	113 (25.3)	40 (21.6)	114 (20.8)	

* Temporary resident indicates the individuals who have household registrations in other regions, and have stayed in Shenzhen city more than 6 months. Floating population indicates the individuals who have household registrations in other regions, and have stayed in Shenzhen less than 6 months

### CD4 T-cell count and plasma HIV RNA load

**Fig. 2:**
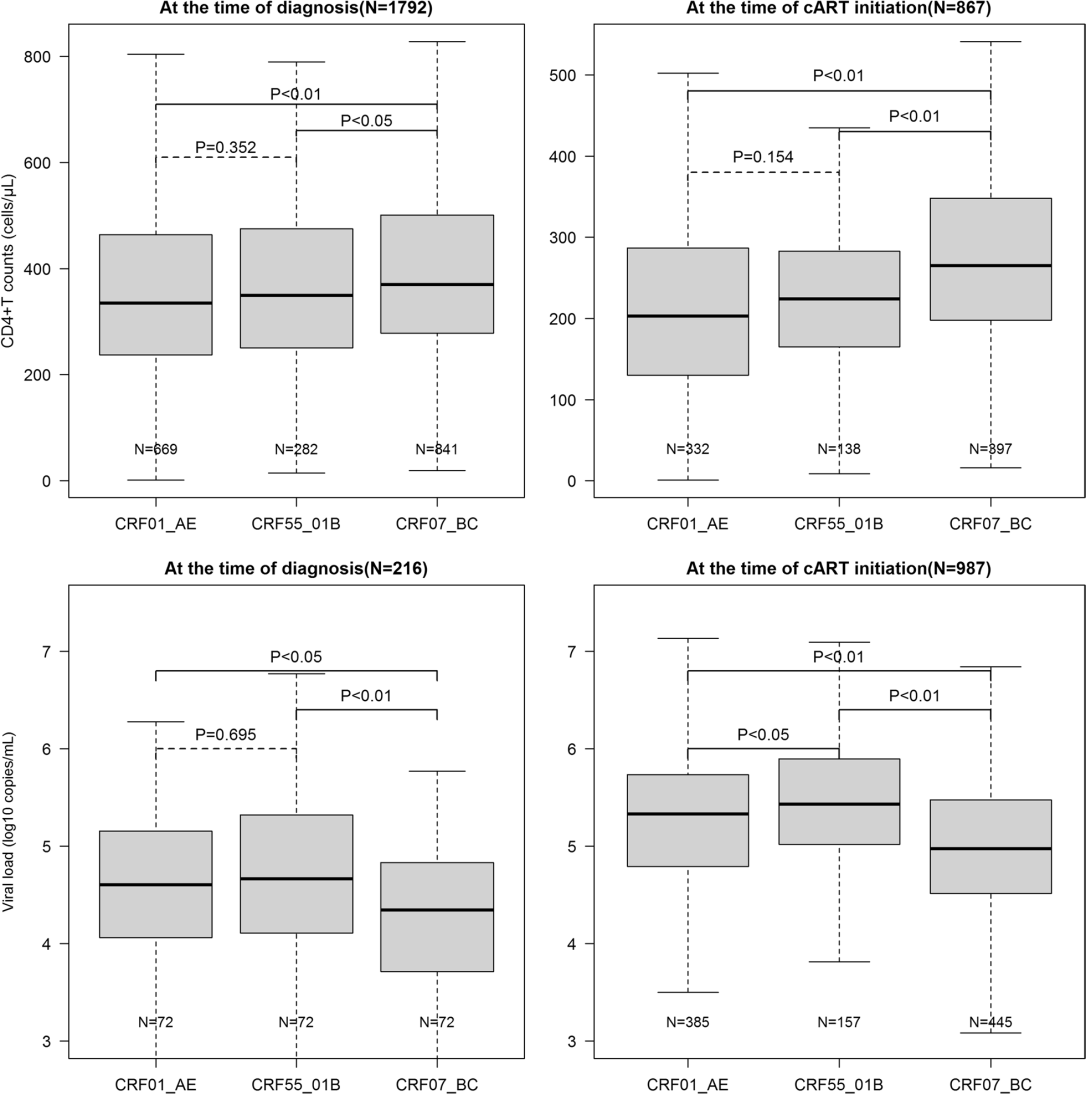
The CD4 count and plasma HIV RNA load at diagnosis and cART initiation

Among the 1792 MSM who were infected with the three main subtypes, CRF55_01B-infected MSM showed a significantly lower median CD4 count than CRF07_BC-infected MSM at diagnosis of HIV infection (349.5 [IQR, 250.2–474.8] vs. 370.0 [IQR, 278.0–501.0], P < 0.05), as well as at initiation of cART (224.0 [IQR, 165.2–283.0] vs. 265.0 [IQR, 198.0–348.0], P < 0.01) (Fig. 2). However, the median CD4 count of CRF55_01B-infected MSM was not significantly different from that of CRF01_AE-infected MSM at diagnosis of HIV infection (349.5 [IQR: 250.2–474.8] vs. 335.0 [IQR: 237.0–464.0], P = 0.352) and at initiation of cART (224.0 [IQR, 165.2–283.0] vs. 203.0 [IQR, 130.5–287.0], P = 0.154). In contrast, CRF55_01B-infected MSM was associated with a significantly higher median plasma HIV RNA load (5.4 log_10_ copies/ml; IQR, 5.0–5.9) than CRF01_AE-infected MSM (5.3 log_10_ copies/ml; IQR, 4.8–5.7, P < 0.05) and CRF07_BC (5.0 log_10_ copies/ml; IQR, 4.5–5.5, P < 0.01) at the initiation of cART (Fig. 2). In addition, those infected with CRF55_01B showed significantly higher plasma HIV RNA load (4.7 log_10_ copies/ml; IQR, 4.1–5.3) than those infected with CRF07_BC (4.3 log_10_ copies/ml; IQR, 3.7–4.8, P < 0.01) at diagnosis, although similar to those infected with CRF01_AE (4.6 log_10_ copies/ml; IQR, 4.1–5.1, P = 0.695).

### Change trend of CD4 T-cell count and plasma HIV RNA load

A total of 753 MSM were included for estimate and comparison of the declining rates of CD4 count by subtype, and 199 MSM were included for analysis of the increasing rates of plasma HIV RNA load by subtype. Demographic characteristics and time interval between the two measurements of CD4 count and viral load were presented in Additional file 1: Tables S1 and S2. Stratified analyses were conducted according to the initial CD4 count at diagnosis as different initial CD4 counts which represent different status of disease progression of the patients. Table 2 showed the estimated declining rates of CD4 counts by subtype. Specifically, for the group with initial CD4 counts between 200 and 350 cells/µl, infection with CRF55_01B was associated with a significantly lower declining rate ([cells/µl]^1/2^/year) than with CRF01_AE (13.4 [95%CI 8.2–18.6] vs. 22.9 [95%CI 15.8–30.0], P < 0.05), while similar to CRF07_BC (13.4 [95%CI 8.2–18.6] vs. 10.8 [95%CI 8.2–13.4], P = 0.383). However, no significant difference was found for the other group with initial CD4 value above 350 cells/µl. Linear mixed effect model results also showed that the declining rate of CD4 count for CRF55_01B-infected MSM was 10.3 [cells/µl]¹/²/year (95%CI 0.7–19.9, P < 0.05) lower than that for CRF01_AE-infected MSM among the patients with CD4 counts of 200–350 cells/µl at HIV diagnosis, with adjustments for age at diagnosis, ethnicity, marital status, and year of HIV diagnosis.

**Table 2:** Estimates of declining rate of CD4 T cell counts from the time of diagnosis to cART initiation

HIV-1 subtype	N	Decline rate (cell/µl)^1/2^ /year)			Decline rate (cell/µl)^1/2^/year)	
		Mean (95%CI)	P value	P value	Adjusted mean (95%CI)*	P value
CD4 200–350						
CRF55_01B	62	13.6 (8.3, 18.9)	Ref		Ref	Ref
CRF01_AE	157	23.3 (15.9, 30.7)	< 0.05	Ref	10.3 (0.7, 19.9)	< 0.05
CRF07_BC	194	11.0 (8.4, 13.5)	0.391	< 0.01	− 2.7 (− 12.0, 6.6)	0.570
CD4 ≥ 350						
CRF55_01B	52	7.9 (3.0, 12.8)	Ref		Ref	Ref
CRF01_AE	114	6.9 (5.0, 8.8)	0.702	Ref	1.4 (− 3.5, 6.2)	0.587
CRF07_BC	174	9.6 (5.6, 13.6)	0.597	0.226	0.7 (− 4.1, 5.5)	0.776
All ≥ 200						
CRF55_01B	114	11.0 (7.3, 14.7)	Ref		Ref	Ref
CRF01_AE	271	16.4 (11.9, 20.9)	0.069	Ref	6.0 (− 0.3, 12.3)	0.064
CRF07_BC	368	10.3 (8.0, 12.7)	0.77	< 0.05	− 0.6 (− 6.7, 5.4)	0.837

* The Adjusted mean was estimated with adjustment of age at HIV diagnosis, ethnicity, marital status, and year of HIV diagnosis

In general, for all MSMS with initial CD4 count of 200 cells/µl or above, CRF55_01B infections caused a significantly higher increasing rate of plasma HIV RNA load than CRF07_BC infections (2.0 log_10_ copies/ml [95%CI 1.1–2.9] vs. 0.7 log_10_ copies/ml [95%CI 0.5–0.9], P < 0.01), while not significantly different from infection with CRF01_AE (1.4 log_10_ copies/ml [95%CI 0.8–2.0], P = 0.324) (Table 3). Furthermore, the model with random effect also showed CRF55_01B infections were 1.2 log_10_ copies/ml/year higher increasing rate of viral load than CRF07_BC infections after adjustment for age at diagnosis, ethnicity, marital status, and year of HIV diagnosis, although not significantly different for the group with initial CD4 count as 350cells/µl or above.

**Table 3:** Estimates of increasing rate of viral load from the time of diagnosis to cART initiation

	N	Increase rate of viral load (log_10_copies/ml/year)			Increase rate of viral load (log_10_copies/ml/year)	
		Mean (95%CI)	P value	P value	Adjusted mean (95%CI)*	P value
CD4 200–350						
CRF55_01B	29	3.5 (1.5, 5.5)	Ref		Ref	Ref
CRF01_AE	33	1.9 (0.9, 2.9)	0.150	Ref	− 1.4 (− 3.2, 0.4)	0.136
CRF07_BC	27	0.9 (0.5, 1.3)	< 0.05	< 0.05	− 2.6 (− 4.5, − 0.7)	< 0.05
CD4 ≥ 350						
CRF55_01B	28	0.9 (0.6, 1.2)	Ref		Ref	Ref
CRF01_AE	21	1.4 (0.8, 2.0)	0.400	Ref	0.2 (− 0.2, 0.7)	0.345
CRF07_BC	28	0.7 (0.5, 0.9)	0.606	0.215	− 0.1 (− 0.5, 0.4)	0.769
All ≥ 200						
CRF55_01B	65	2.0 (1.1, 2.9)	Ref		Ref	Ref
CRF01_AE	65	1.4 (0.8, 2.0)	0.324	Ref	− 0.5 (− 1.5, 0.4)	0.250
CRF07_BC	69	0.7 (0.5, 0.9)	< 0.05	< 0.05	− 1.2 (− 2.1, − 0.3)	< 0.01

* The Adjusted mean was estimated with adjustment of age at HIV diagnosis, ethnicity, marital status, and year of HIV diagnosis

## Discussion

Our study found that CRF55_01B, a newly emerged CRF of HIV-1, had become the third predominant strain in recent years in Shenzhen, China. With long-term surveillance data of the past 10 years in Shenzhen, this study for the first time revealed the impact of infection with CRF55_01B on the immunological progression and viral replication in the cART-naive MSM and compared it with other two dominant subtypes. We found that CRF55_01B-infected MSM had significantly lower CD4 count than CRF07_BC-infected MSM both at diagnosis and the initiation of cART. Furthermore, CRF55_01B showed significantly slower loss of CD4 count than CRF01_AE among the patients with initial CD4 count of 200–350 cells/µl. Meanwhile, CRF55_01B-infected MSM showed significantly higher plasma HIV RNA load than MSM infected with CRF01_AE or CRF07_BC at the initiation of cART. Moreover, infection with CRF55_01B was associated with a significantly faster increase of plasma HIV RNA load than infection with CRF01_AE and CRF07_BC, especially among the patients with initial CD4 count of 200–350 cells/µl.

CRF55_01B had overtaken subtype B as the third predominant strain in Shenzhen since 2012. Recent studies indicated that CRF55_01B had rapidly spread and epidemic from Guangdong-Shenzhen to other provinces of China with prevalence ranging from 1.5% to 12.5% since 2010; of note, the highest prevalence was reported in Guangdong, China [12–14]. Our study revealed that infection with CRF55_01B had significantly slower loss of CD4 count than infection with CRF01_AE among those with the initial CD4 count between 200 and 350 cells/µl. Slow depletion of CD4 T-cell usually indicates slow immunological progression and less impairment to human system, which may translate to a prolonged asymptomatic state and increased risk for onward HIV transmission [15]. Although the significant difference was only observed in patients with initial CD4 count between 200 and 350 cells/µl, it was justifiable because previous study demonstrated that only baseline CD4 count below 350 cells/µl was significantly associated with rapid decline of CD4 count [16]. A recent nationwide HIV molecular epidemiologic survey by China CDC discovered that people infected with CRF01_AE cluster 5 had significantly higher CD4 count than those infected with CRF01_AE cluster 4 [17]; meanwhile, our unpublished data based on general population found that CRF55_01B-infected MSM had even higher CD4 count than those infected with CRF01_AE cluster 5, which may be attributed to the slower loss of CD4 count caused by infection with CRF55_01B than with CRF01_AE. More studies are warranted to confirm our findings.

It is well documented that patients infected with CRF01_AE had higher plasma HIV RNA load than those with CRF07_BC [18]. A previous cohort study suggested that CRF07_BC was associated with relatively lower plasma HIV RNA load, which could probably be explained by its poorer protease-mediated processing and slower viral maturation processes [19]. However, our study found that patients infected with CRF55_01B had even higher plasma HIV RNA load than those infected with CRF01_AE at the initiation of cART, and had faster increasing rate than those infected with CRF01_AE during the rapid disease progression period. Higher viral load and faster increasing rate of viral load represent higher viral replication capacity, and may increase the risk of HIV transmission [20]. Thus, the faster increase of viral load together with the relatively slower loss of CD4 T-cell may help to explain the recent surging dominance and continued expansion of CRF55_01B among MSM in China. As the published data on CRF55_01B were only limited to the genomic and evolutionary characteristics, information on its impact on the disease progression has rarely been reported, making it difficult to compare our findings with studies from other regions.

Consistent with a recent study from Guangdong, our study found CRF07_BC and CRF01_AE were the two predominant subtypes in Shenzhen [21]. CRF01_AE, first imported to China in 1994 [22], now has become a main strain in MSM in several cities of China [23–25], and highly dynamic in Southeast Asia [26]. The predominant HIV-1 epidemic strain in the Chinese MSM has recently changed from subtype B of US-European origin in 2005 to CRF01_AE in 2012 [27]. Previous study suggested that CRF01_AE strain with a high frequency of CXCR4-tropism circulating in MSM population might cause a severe loss of CD4 count and speed up the disease progression, compared to CRF07_BC and subtype B strains [8]. Other studies also found that CRF01_AE strains was associated with a higher annual rate of CD4 loss and accelerated disease progression than the non-CRF01_AE strains [28, 29].

Infection with CRF55_01B was associated with lower CD4 count than infection with CRF07_BC both at diagnosis and the initiation of cART in this study. CRF07_BC, the generation of Thailand variant of subtype B and Indian subtype C, was initially found among IDUs in southwest China in the 1990’s and then spread to other areas [30–32]. It was first found to be transmitted to MSM since 2004 and has become one of the most prevalent strains for over ten years [13, 33]. A study from Taiwan suggested that CRF07_BC among IDUs was associated with relatively slower depletion of CD4 count, which was attributed to the 7-amino acid deletion observed in p6 of CRF07_BC [34].

It is also noteworthy that previous molecular epidemiologic analysis demonstrated that CRF55_01B might be originated from MSM in Shenzhen and later transmitted to heterosexual population, and then spread to other regions of China through the migrant MSM [6, 14]. As the largest immigration city in China, Shenzhen is a popular gathering place for migrant MSM from all over the country, Shenzhen would serve an important hub in the ongoing outspreading of CRF55_01B to other regions in China as well as other countries. A recent report of CRF55_01B infection in Hangzhou might be a prelude of outbreak in the central eastern China in the near future [8]. What’s more, several new unique recombinant strains (e.g., SZ44LS7251 in Shenzhen) derived/originated from CRF55_01B and CRF07_BC were reported in southern China [5, 35, 36], which may further complicate the genomic structures and give rise to super-infections or co-infections in the MSM community. All these potential consequences together pose serious challenges to the prevention and control of HIV infection, as well as the treatment and vaccine development [37]. Therefore, in-depth study and timely prevention and control of CRF55_01B infection are needed for both treatment strategies and prevention of potential epidemic.

There are several limitations worth noting. First, our study lack the estimated date of seroconversion of infected MSM, a common limitation in studies on disease progression [38]. Thus, the change rates for CD4 count and plasma HIV RNA load were not observed from the time of infection, but from the time of diagnosis. Nevertheless, we had adjusted the estimates by divide the dataset into three subsets with different initial level of CD4 counts, which could provide a rough proxy for different stages of disease progression. Secondly, this study only obtained two determinations of CD4 count and plasma HIV RNA load at diagnosis and the initiation of cART; thus, it is difficult to evaluate the dynamic change over time. Further study with more repeated measurements would be desirable. Also, the clinical outcomes should be monitored. Thirdly, the sample size for MSM with measurement on viral load at diagnosis was relatively small, which may lead to limited statistical power. Thus, future cohort studies with a larger sample size and more repeated measurements are needed to validate our findings.

## Conclusions

This study elucidated the pathogenic, immunologic and virological characteristics of HIV-1 CRF55_01B in infected MSM who were prior to cART. The relatively slower loss of CD4 count together with faster increase of plasma HIV RNA load suggest that CRF55_01B may prolong the asymptomatic phase and increase the risk of HIV transmission, which would favor further rise and potential epidemic in the near future. Our findings are helpful in understanding the disease progression and the recent surge of CRF55_01B among MSM in China, providing significant implications for prevention and interventions to forestall potential epidemic and outspreading of CRF55_01B to other areas in China or other countries.

## Methods

### Study subjects

HIV-1-infected MSM who were newly diagnosed with recent HIV infection and had been living in Shenzhen from 2005 to 2015 were screened according to the following conditions: (1) HIV-1 infection confirmed by Western blot (HIV blot 2.2, MP Diagnostics, Singapore); (2) HIV transmission through male-to-male sex; (3) prior to cART; (4) *pol* genes being sequenced and phylogenetically identified as CRF55_01B, CRF01_AE or CRF07_BC. Recent HIV infection was defined as those with fraction of ambiguous nucleotides lower than 0.5% in *pol* genes, which provides strong evidence against an infection event < 1 year prior to sampling [39]. Written informed consent was obtained from all the participants recruited in this project, and the demographic data (such as gender, age, marital status, etc.) were also collected through face-to-face questionnaire interview. The protocol for enrollment of MSM in this study was approved by the Medical Ethics Committee of Shenzhen Center for Disease Control and Prevention (CDC).

Samples were collected at diagnosis and initiation of cART from patients seeking HIV care at Shenzhen Center for Disease Control and Prevention and the Third People’s Hospital of Shenzhen. Each blood sample was collected in two EDTA- Vacutainer tubes with 5 ml/tube (Becton and Dickinson Company, USA). One tube of whole blood sample was for quantification of CD4 count; and the other tube was centrifuged at 1500*g* for 10 min at room temperature to separate plasma and buffy coat. Plasma was frozen in multiple aliquots at − 80 °C until use.

### Phylogenetic analysis

HIV-1 genome RNA was extracted from stored plasma specimens using the QIAmp Viral RNA Mini kit (Qiagen, Valencia, CA, USA) as manufacturer’s instructions. Fragment of *pol* (from 2253 to 3314 according to HXB2 calibrator) spanning the protease gene and partial reverse transcriptase gene were amplified by reverse transcription and nested PCR and then sequenced [40], with sets of primers and thermal cycling conditions as described previously [41]. Each sequence was blasted through the Los Alamos HIV-1 database, and checked for the existence of ambiguous nucleotides. The resulting gene fragment sequences were aligned with reference sequences to determine the subtypes while the phylogenetic tree was constructed by the neighbor-joining method implemented by MEGA version 7.0. Mean genetic distance of CRF01_AE, CRF07_BC and CRF55_01B strains was calculated under P-distance model (bootstrap value = 500) using MEGA 7.0.

### CD4 T-Cell count and viral load assay

CD4 count were measured in our laboratory by flow cytometry (FACS Calibur, Becton and Dickinson BD Company, USA) within 24 h according to the instructions of BD Tritest CD4/CD8/CD3 (FITC/PE/PerCP, Becton and Dickinson Company, USA). HIV-1 genome RNA was extracted from stored plasma specimens using the HIV-1 nucleic acid testing of fluorescent PCR kit (Daan Gene, Guangzhou, China). PCR was performed on ABI7500 with quality control and calibrator, and viral load was automatically calculated as manufacturer’s instructions. The linear detection range was 5.0 × 10^2^ to 1.0 × 10^8^ copies/ml.

### Statistical analysis

Demographic information of infections with different subtypes was tested for statistical difference by Chi-square test (χ^2^-test) or Fisher’s exact test where applicable. Plasma HIV-1 RNA load and CD4 counts of patients infected with different HIV-1 subtypes (CRF01_AE, CRF07_BC, and CRF55_01B) were described using median and interquartile range (IQR). Kruskal-Wallis test (multiple independent samples nonparametric test) and Mann-Whitney test (two independent samples nonparametric test) were used to compare the CD4 counts and viral load among patients infected with different subtypes.

As previous studies suggested that serial CD4 observations decayed approximately linearly on the square root scale, square root transformation of CD4 value was applied to linearize the changes from the time of diagnosis to cART initiation in this study [10]. Log_10_-transformation of viral load was utilized to linearize the change rate over time as well. Multilevel linear mixed effect models was used to compare the annual rate of CD4 cell decline and viral load increase. The final models adjusted for age at HIV diagnosis, ethnicity, marital status, and year of HIV diagnosis. As the date of infection was not known in this study, a random effect of the initial CD4 cell count was included in the models. Year of HIV diagnosis was also include as a categorical random effect to account for the random deviations of the criteria for cART initiation in the past years. Various studies on the decline of CD4 cell counts suggest those first CD4 cell measurement made in the acute infection phases should be excluded. In this study, those with first CD4 cell count lower than 200 cells/µl or only have one measurement of CD4 cell count were excluded from the analysis for comparison of CD4 cell count declining and viral load increase by subtype.

All statistical tests were 2-tailed, probability value < 0.05 were considered statistically significant. All statistical analyses were performed in R (version 3.4.4, Foundation for Statistical Computing, Vienna, Austria).

## Supplementary Information

**Additional file 1: Table S1.** Demographic characteristics of the 753 MSM with twice measurements of CD4 T-cell counts. **Table S2.** Demographic characteristics of the 216 MSM with twice measurements of HIV RNA viral load.

## Data Availability

The datasets supporting the conclusions of this article are included within the article and its additional files.

## Abbreviations

CRFs: Circulating recombinant forms
URFs: Unique recombinant forms
MSM: Men who have sex with men
CDC: Center for Disease Control and Prevention
IQR: Interquartile range
