# Supplementary material for: Impact of HIV-1 CRF55_01B infection on the evolution of CD4 count and plasma HIV RNA load in men who have sex with men prior to antiretroviral therapy
Source: Retrovirology. 2021 Aug 16;18:22. doi: 10.1186/s12977-021-00567-z (PMC8365277; doi:10.1186/s12977-021-00567-z)
Supplement: Supplementary file 1 — Additional file 1: Table S1. Demographic characteristics of the 753 MSM with twice measurements of CD4 T-cell counts. Table S2. Demographic characteristics of the 216 MSM with twice measurements of HIV RNA viral load. [file 12977_2021_567_MOESM1_ESM.docx]

Table S1 Demographic characteristics of the 753 MSM with twice measurements of CD4 T-cell counts

| N=753 | CRF01_AE | CRF55_01B | CRF07_BC | P value |
| --- | --- | --- | --- | --- |
|  | N=271 | N=114 | N=368 |  |
| **Year** |  |  |  |  |
| 2005-2008 | 11(4.1) | 3(2.6) | 11(3.0) | 0.870 |
| 2009 | 17(6.3) | 4(3.5) | 12(3.3) |  |
| 2010 | 6(2.2) | 5(4.4) | 12(3.3) |  |
| 2011 | 27(10.0) | 8(7.0) | 32(8.7) |  |
| 2012 | 51(18.8) | 26(22.8) | 72(19.6) |  |
| 2013 | 64(23.6) | 27(23.7) | 86(23.4) |  |
| 2014 | 50(18.5) | 23(20.2) | 69(18.8) |  |
| 2015 | 45(16.6) | 18(15.8) | 74(20.1) |  |
| **Age(year)** |  |  |  |  |
| <25 | 85(31.4) | 35(30.7) | 103(28.0) | 0.426 |
| 26-35 | 133(49.1) | 47(41.2) | 170(46.2) |  |
| 36-45 | 39(14.4) | 22(19.3) | 72(19.6) |  |
| >46 | 14(5.2) | 10(8.8) | 23(6.2) |  |
| **Marital status** |  |  |  |  |
| Unmarried | 219(80.8) | 72(63.2) | 273(74.2) | <0.01 |
| Married | 36(13.3) | 33(28.9) | 65(17.7) |  |
| Divorced or widowed | 16(5.9) | 6(5.3) | 30(8.2) |  |
| Unknown | 0(0.0) | 3(2.6) | 0(0.0) |  |
| **Enthncity** |  |  |  |  |
| Han | 262(96.7) | 107(93.9) | 351(95.4) | 0.422 |
| Non-han | 9(3.3) | 7(6.1) | 17(4.6) |  |
| **Education** |  |  |  |  |
| Below college or university | 63(23.2) | 44(38.6) | 109(29.6) | <0.05 |
| College or university | 106(39.1) | 28(24.6) | 133(36.1) |  |
| Unknown | 1(0.3) | 0(0.0) | 0(0.0) |  |
| **Census registration** |  |  |  |  |
| Shenzhen | 22(12.5) | 8(11.0) | 25(11.1) | 0.586 |
| Temporary stay | 114(64.8) | 52(71.2) | 162(72.0) |  |
| Floating or unknown | 40(22.7) | 13(17.8) | 38(16.9) |  |
| **Time interval(year)** | 0.4(0.1,1.8) | 0.6(0.1,1.5) | 0.5(0.1,1.6) | 0.516 |

Note:*Temporary resident indicates the individuals who have household registrations in other regions, and have stayed in Shenzhen city more than six months. Floating population indicates the individuals who have household registrations in other regions, and have stayed in Shenzhen less than six months.

Table S2 Demographic characteristics of the 216 MSM with twice measurements of viral load

| N=199 | CRF01_AE | CRF55_01B | CRF07_BC | P value |
| --- | --- | --- | --- | --- |
|  | N=65 | N=65 | N=69 |  |
| **Year** |  |  |  |  |
| 2010 | 2(3.1) | 3(4.6) | 3(4.3) | 0.997 |
| 2011 | 5(7.7) | 7(10.8) | 6(8.7) |  |
| 2012 | 17(26.2) | 12(18.5) | 16(23.2) |  |
| 2013 | 21(32.3) | 23(35.4) | 21(30.4) |  |
| 2014 | 17(26.2) | 16(24.6) | 19(27.5) |  |
| 2015 | 3(4.6) | 4(6.2) | 4(5.8) |  |
| **Age(year)** |  |  |  |  |
| <25 | 19(29.2) | 19(29.2) | 19(27.5) | 0.451 |
| 26-35 | 32(49.2) | 28(43.1) | 29(42.0) |  |
| 36-45 | 12(18.5) | 11(16.9) | 18(26.1) |  |
| >46 | 2(3.1) | 7(10.8) | 3(4.3) |  |
| **Marital status** |  |  |  |  |
| Unmarried | 53(81.5) | 40(61.5) | 50(72.5) | 0.202 |
| Married | 10(15.4) | 20(30.8) | 16(23.2) |  |
| Divorced or widowed | 2(3.1) | 4(6.2) | 3(4.3) |  |
| Unknown | 0(0.0) | 1(1.5) | 0(0.0) |  |
| **Ethnicity** |  |  |  |  |
| Han | 63(96.9) | 61(93.8) | 67(97.1) | 0.665 |
| Non-han | 2(3.1) | 4(6.2) | 2(2.9) |  |
| **Education** |  |  |  |  |
| Below senior high school | 16(24.6) | 23(35.4) | 21(30.4) | 0.174 |
| Senior high school or technical secondary school | 19(29.2) | 24(36.9) | 28(40.6) |  |
| College or university | 30(46.2) | 18(27.7) | 20(29.0) |  |
| **Census registration** |  |  |  |  |
| Shenzhen | 6(13.3) | 6(13.3) | 4(8.7) | 0.209 |
| Temporary stay | 29(64.4) | 33(73.3) | 39(84.8) |  |
| Floating or unknown | 10(22.2) | 6(13.3) | 3(6.5) |  |
| **Time interval (year)** | 0.8(0.5,1.5) | 1(0.3,1.6) | 1(0.7,1.4) | 0.214 |

Note:*Temporary resident indicates the individuals who have household registrations in other regions, and have stayed in Shenzhen city more than six months. Floating population indicates the individuals who have household registrations in other regions, and have stayed in Shenzhen less than six months.
